# A registry-based observational study comparing emergency calls assessed by emergency medical dispatchers with and without support by registered nurses

**DOI:** 10.1186/s13049-021-00987-y

**Published:** 2022-01-10

**Authors:** Klara Torlén Wennlund, Lisa Kurland, Knut Olanders, Maaret Castrén, Katarina Bohm

**Affiliations:** 1 Department of Clinical Science and Education, Södersjukhuset, Karolinska Institutet, 118 83 Stockholm, Sweden; 2 Department of Medical Sciences and Department of Emergency Medicine, Örebro University, 70181 Örebro, Sweden; 3 Department of Intensive and Perioperative Care, Skåne University Hospital, Lund, Sweden; 4 Department of Emergency Medicine, Helsinki University, Helsinki, Finland; 5 Department of Emergency Medicine and Services, Helsinki University Hospital, Helsinki, Finland; 6 Emergency Department, Södersjukhuset, Stockholm, Sweden

**Keywords:** Emergency medical dispatch, Emergency medical dispatcher, Registered nurse, Emergency Medical Communication Center, Emergency medical services, Pre-hospital triage

## Abstract

**Background:**

The requirement concerning formal education for emergency medical dispatcher (EMD) is debated and varies, both nationally and internationally. There are few studies on the outcomes of emergency medical dispatching in relation to professional background. This study aimed to compare calls handled by an EMD with and without support by a registered nurse (RN), with respect to priority level, accuracy, and medical condition.

**Methods:**

A retrospective observational study, performed on registry data from specific regions during 2015. The ambulance personnel’s first assessment of the priority level and medical condition was used as the reference standard. Outcomes were: the proportion of calls dispatched with a priority in concordance with the ambulance personnel’s assessment; over- and undertriage; the proportion of most adverse over- and undertriage; sensitivity, specificity and predictive values for each of the ambulance priorities; proportion of calls dispatched with a medical condition in concordance with the ambulance personnel’s assessment. Proportions were reported with 95% confidence intervals. χ^2^-test was used for comparisons. P-levels < 0.05 were regarded as significant.

**Results:**

A total of 25,025 calls were included (EMD n = 23,723, EMD + RN n = 1302). Analyses relating to priority and medical condition were performed on 23,503 and 21,881 calls, respectively. A dispatched priority in concordance with the ambulance personnel’s assessment were: EMD n = 11,319 (50.7%) and EMD + RN n = 481 (41.5%) (*p* < 0.01). The proportion of overtriage was equal for both groups: EMD n = 5904, EMD + RN n = 306, (26.4%) *p* = 0.25). The proportion of undertriage for each group was: EMD n = 5122 (22.9%) and EMD + RN n = 371 (32.0%) (*p* < 0.01). Sensitivity for the most urgent priority was 54.6% for EMD, compared to 29.6% for EMD + RN (*p* < 0.01), and specificity was 67.3% and 84.8% (*p* < 0.01) respectively. A dispatched medical condition in concordance with the ambulance personnel’s assessment were: EMD n = 13,785 (66.4%) and EMD + RN n = 697 (62.2%) (*p* = 0.01).

**Conclusions:**

A higher precision of emergency medical dispatching was not observed when the EMD was supported by an RN. How patient safety is affected by the observed divergence in dispatched priorities is an area for future research.

**Supplementary Information:**

The online version contains supplementary material available at 10.1186/s13049-021-00987-y.

## Background

Emergency medical dispatching is the first link in the chain of emergency care [1, 2]. An assessment of the urgency and the nature of the caller’s medical situation is performed by the emergency medical dispatcher (EMD) when an emergency medical call is made. A high level of precision of emergency medical dispatching—i.e., the dispatching of a timely response with the appropriate resource (s)—is important for ensuring both patient safety and the best use of resources. Currently, the requirements for formal education for EMDs vary among emergency medical services both nationally and internationally [3–7], and few studies have investigated either the optimal education for an EMD [8–10] or the relationship between educational and professional backgrounds of EMDs and the outcomes of emergency medical dispatching [11–13].

The Swedish emergency call number (112) is operated by a national, publicly owned organization with 15 Emergency Medical Communication Centers (EMCC) nationwide. The organization is responsible for emergency medical dispatching in the majority of the Sweden’s regions [14, 15]. All emergency calls are primarily handled by a certified EMD. EMDs are not required to have medical training in Sweden but are certified after a 14-week training program and are also required to perform an including annual re-certification. When the EMD identifies a medical emergency, then precedes to apply the criteria-based dispatch protocol, the “Swedish Index to Emergency Medical Assistance” (Swedish Index), to decide whether and with which priority an ambulance should be dispatched to the caller [16]. The EMD can request support from a registered nurse (RN), to assist in the dispatching decision [14]. The request for assistance is made electronically and the first available RN at any of the national EMCCs will respond. The RN can either have an advisory and supportive function or take over the call. However, whether and how the support by an RN affects the outcome of the emergency medical dispatch has not previously been investigated. Therefore, the aim of the current study was to compare calls handled by an EMD with and without RN support with respect to priority level, accuracy, and medical condition.

## Methods

### Study design

A retrospective observational study was performed January 1 to June 14, 2015, based on registry data from SOS Alarm. The assessment of priority level and medical condition of the patient by the ambulance personnel was used as the reference standard. Outcomes of calls handled by an EMD alone and calls when the EMD was supported by an RN were evaluated and compared.

Data was collected from a quality improvement project involving SOS Alarm and specific regions and ambulance enterprises with the objective of linking ambulance personnel’s assessment to dispatching, which had not routinely been done in Sweden. For this time limited project, the ambulance personnel assessed the patient in accordance with the triage scale “Rapid Emergency Triage and Treatment System” (RETTS) [17] before further care was administered, and reported the information back to SOS Alarm with the help of a computer-assisted system (a process evaluated previously [18]).

### Study setting

The catchment area of the ambulances participating in the quality improvement project included urban, suburban, and rural settings and were, at the time of data collection, home for almost 1.5 of Sweden’s 9.9 million inhabitants (Table 1).

**Table 1:** Ambulances participating in the quality improvement project and their catchment areas in 2015

Region	No. of ambulance stations and ambulances	Covering km^2^ (No. of inhabitants)
Halland	8 Stations, 29 ambulances	5454 (314,784)
Jönköping	10 Stations, 21 ambulances	10,475 (344,752)
Skåne	13 Stations, 31 ambulances	6890 (490,666)
Örebro	9 Stations, 16 ambulances	8546 (291,000)

Approximately 3 million calls are made to 112 each year, one third of which are medical emergencies. An EMD at any of the national EMCCs, runed by a publicly owned organization (SOS Alarm) assesses each call. The EMD assigns a dispatch priority level and a medical condition, referring to the level of urgency and the nature of the caller’s symptoms. If it is determined that an ambulance is required an ambulance dispatcher dispatches an ambulance accordingly [19, 20].

Medical professionals are employed at the EMCC to provide additional medical support. The EMD can request the support of an RN (or occasionally, due to regional agreements, a physician), to assist in evaluating dispatch priority and medical condition [14, 21]. Prior to and during the study period, there were no predefined criteria for the type of calls or in which situations RN support should be requested—at that time, this decision was left to the clinical judgement of the individual EMD. The RN can either support the EMD directly by connecting to the phone call, which enable the RN to speak to the caller or support the EMD through a chat function. In either case, once they are involved in an emergency call, the RN are responsible for the dispatch priority and medical condition.

The educational requirements for an EMD are a high school degree and the ability to speak fluent Swedish and English. All EMDs undergo a 14-week program. The program includes education, training, and certification, and annual re-certification is required [21]. The educational requirement for an RN in Sweden is a bachelor’s degree in Nursing Science, comprising three years of university studies. RNs also undergo a training and certification program upon employment at SOS Alarm.

The criteria-based dispatch protocol the “Swedish Medical Index to Emergency Medical Assistance” (Swedish Index) is used as the decision support tool [16, 19]. The procedure according to this protocol is that first, the EMD assesses the vital signs (e.g. breathing and level of consciousness) and determines the geographical position of the caller. The EMD then selects one of the 30 chapters, each referring to a chief complaint, that is closest to the as presented by the patient. A subdivision in each chapter lists different medical conditions, including a more detailed symptom description and criteria that has the purpose of guiding the EMD to assign the appropriate priority level: from Priority 1 (the most urgent, ongoing threat to life) to Priority 3 (least urgent). Priority 4 is assigned to patients not requiring medical assistance but in need of assistance for transportation to a health care facility [16, 19].

In accordance with Swedish legislation, all ambulances are staffed with at least one RN, typically with a year of specialist training in prehospital care, who has the medical responsibility for the patient [15, 22]. The ambulance personnel use triage scales to assess the acuity of the patient. The Rapid Emergency Triage and Treatment System (RETTS) is the most common triage scale used in the ambulance and in hospital emergency departments in Sweden [17, 23–27]. RETTS is a color-graded priority scale, spanning from Red (most urgent, ongoing threat to life) to Orange, Yellow and Green (least urgent) and Blue (non-ambulance mission). RETTS consists of an algorithm for vital signs (airway, breathing, circulation, mental status, and environment) on presentation and flowcharts for emergency signs and symptoms (ESS) reflecting the patient’s symptomology on presentation. When combined, these generate the priority [17].

### Study material

Inclusion criteria for the current study were emergency medical calls resulting in ambulance dispatch. Exclusion criteria were calls when the ambulance personnel did not assess the patient on the scene, or when the assessment by the ambulance personnel was inconclusive. Partial data was excluded in cases when multiple ambulances were dispatched to and reported on the same patient, and information from a given case was reported only once.

Analyses relating to priority were performed on calls with complete and corresponding data on dispatched priority (Swedish Index Priority 1–3) and assessment by ambulance personnel (RETTS Red/Orange/Yellow/Green). Analyses relating to medical condition were performed on calls with complete and corresponding data on dispatched medical condition (Swedish Index) and ambulance personnel’s assessment of medical condition (RETTS ESS).

### Definitions

The ambulance personnel’s assessment of the priority and medical condition, according to RETTS, was used as the reference standard in the analyses. The corresponding priorities and medical conditions in the Swedish Index and RETTS were defined prior to analysis and based on predefined criteria for each scale (priorities, Table 2; medical conditions, Additional file 1) [16, 17]. The Swedish Index and RETTS have unequal numbers of priority levels. The two highest priorities in RETTS were therefore merged into one single priority (Red/Orange), defined as that corresponding to the highest priority in the Swedish Index (Priority 1) to allow for comparisons.

**Table 2:** Definition of the corresponding priorities in the Swedish Index and RETTS and definitions of over/undertriage

	Ambulance priority level (RETTS)*		
	Red or Orange	Yellow	Green
Dispatched priority level (Swedish Index)**			
1	CORRESPONDING	Overtriage	Overtriage
2	Undertriage	CORRESPONDING	Overtriage
3	Undertriage	Undertriage	CORRESPONDING

Swedish Index, The Swedish Index to Emergency Medical Assistance; RETTS, The Rapid Emergency Triage and Treatment System

*RETTS, predefined criteria for each priority level:

**Swedish Index, predefined criteria for each priority level:

(1) Acute ongoing life-threatening condition, ambulance dispatched with lights and sirens

(2) Acute condition, less time-sensitive than priority level 1

(3) Non-urgent conditions

(Red) Life-threatening condition, need for immediate medical assistance

(Orange) Potentially life-threatening condition, need for immediate medical assistance

(Yellow) Severe, non-life threatening condition, immediate medical assistance can wait

(Green) Non-life threatening condition, less urgent than Yellow, medical assistance can wait

Over- and undertriage were defined as the dispatched priority being higher or lower respectively than the priority as assessed by the ambulance personnel (Table 2). The most adverse over-triage was defined as when a call dispatched as Priority 1 (most urgent) was assessed by the ambulance personnel as RETTS Green (least urgent). The most adverse undertriage was defined as when a call dispatched as Priority 3 was assessed by the ambulance personnel as RETTS Red/Orange (most urgent) (Table 2).

Accuracy was defined as the sensitivity, specificity and predictive values (positive predictive value, PPV; negative predictive value, NPV) for each of the ambulance priorities (RETTS Red/Orange or Yellow or Green) compared to the corresponding dispatch priorities (Swedish Index Priority 1 or Priority 2 or Priority 3) (Table 3).

**Table 3:** Definition of accuracy: Sensitivity, specificity and predictive values for each RETTS priority

		Ambulance priority level (RETTS: Red/Orange* or Yellow or Green)		
		Positive	Negative	
Dispatched priority level (Swedish Index: Priority 1 or Priority 2 or Priority 3)	Positive	a	b	Positive Predictive Value PPV = a/(a + b)
	Negative	c	d	Negative Predictive Value NPV = d/(c + d)
		Sensitivity = a/(a + c)	Specificity = d/(b + d)	

Accuracy was defined as the sensitivity, specificity and predictive values for each of the ambulance priorities (RETTS Red/Orange or Yellow or Green) compared to the corresponding dispatch priorities (Swedish Index Priority 1 or Priority 2 or Priority 3)

Swedish Index, The Swedish Index to Emergency Medical Assistance; RETTS, The Rapid Emergency Triage and Treatment System

*The Swedish Index and RETTS have unequal numbers of priority levels. The two highest priorities in RETTS were therefore merged into one single priority (Red/Orange), defined as that corresponding to the highest priority in the Swedish Index (Priority 1) to allow for comparisons

### Statistical analysis

Calls handled only by an EMD and calls when an RN supported the EMD (EMD + RN) were compared with respect to the following: the proportion of calls dispatched with a priority in concordance with the ambulance assessment; the proportion of over- and undertriage; the proportion of most adverse over- and undertriage; sensitivity, specificity and predictive values for each of the ambulance priorities (RETTS Red/Orange, Yellow and Green); proportion of calls dispatched with a medical condition in concordance with the ambulance personnel’s assessment.

For categorical variables, proportions were reported with 95% confidence intervals (95% CI) and χ^2^-test was used for comparisons between groups. P-levels < 0.05 were regarded as significant. All analyses were performed using SPSS statistical software, version 25 [28].

### Ethical considerations

The current study has been approved by the Swedish Regional Ethical Board in Stockholm, project nr: 2008/810-31/2 and 2012/1419-32.

## Results

### Study cohort

**Fig. 1:**
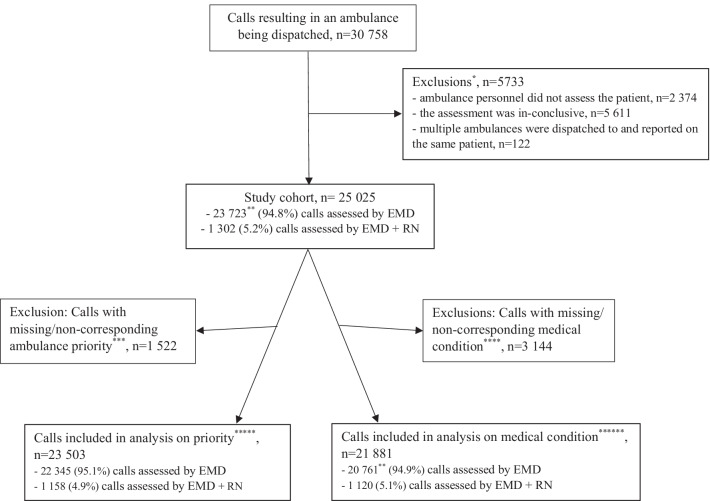
Flow chart illustrating study cohort and exclusions. Abbreviations: Emergency medical dispatcher (EMD), Registered nurse (RN), "Swedish Index to Emergency Medical Assistance" (Swedish Index), "The Rapid Emergency Triage and Treatment System" (RETTS), "Emergency Signs and Symptoms" (ESS). *Caller declined care: n = 1295, Ambulance mission not completed: n = 101, Caller not found: n = 552: Assisting unit: n = 426, Ambulance assessment reported as “Same assessment as the dispatcher”: n = 3237. **In one (n = 1) call the EMD requested the support by an RN, but the request was not fulfilled/answered. ***Reported ambulance priority missing: n = 133. Non-corresponding ambulance priority reported: Child n = 1116, Blue n = 60, Caller dead at scene n = 213. ****Reported ambulance medical condition missing, n = 2445. Dispatched medical condition missing, n = 13. Non-corresponding medical conditions: Child-disease n = 190, Animal/Insect-bite n = 4, Hypo/Hyperthermia n = 3, Danger of life n = 1, Child-unconscious n = 14, Adult unconscious, n = 445, Suspected health service demand, n = 29. *****Calls with complete data on dispatched priority (Swedish Index Priority 1–3) and assessment by ambulance on priority (RETTS Red/Orange/Yellow/Green). ******Calls with complete and corresponding data on dispatched medical condition (Swedish Index) and ambulance assessment of medical condition (RETTS ESS)

The total number of calls resulting in an ambulance being dispatched during the study period was 30,758. After inclusion and exclusion criteria were applied, 25,025 calls remained for the final analysis (Fig. 1). Of these, 23,723 (94.8%) calls were assessed by an EMD and 1,302 (5.2%) by an EMD + RN. Of the included calls, 23,503 (97.8%) calls had complete and corresponding dispatch and ambulance data about priority and 21,881 (91.2%) calls had complete and corresponding dispatch and ambulance data about medical condition (Fig. 1).

### Proportion of calls dispatched with a priority in concordance with the ambulance personnel’s assessment and levels of over/undertriage

The distributions of dispatched priority in relation to the priority assessed by the ambulance personnel, for calls assessed by an EMD and an EMD + RN respectively, are presented in Table 4. The EMD dispatched the calls as Priority 1: 9,502 (42.5%); Priority 2: 11,640 (52.1%); Priority 3: 1,203 (5.4%). EMD + RN dispatched the calls as Priority 1: 231 (19.9%); Priority 2: 732 (63.4%); Priority 3: 193 (16.7%).

**Table 4:** Proportion of calls dispatched with a priority in concordance with the ambulance personnel’s assessment, and proportions of over/undertriage

	n (%)*		Ambulance priority level, RETTS								Dispatched priority in concordance with ambulance personnel's assessment		Overtriage		Undertriage	
			Red		Orange		Yellow		Green							
*Dispatched priority level, Swedish Index*																
1																
EMD	9502 (42.5)		1809		3659		3005		1029		5468		4034		X	
EMD + RN	231 (19.9)		38		76		86		31		114		117		X	
2																
EMD	11,640 (52.1)		860		3379		5531		1870		5531		1870		4239	
EMD + RN	734 (63.4)		35		192		318		189		318		189		227	
3																
EMD	1203 (5.4)		58		247		578		320		320		X		883	
EMD + RN	193 (16.7)		10		34		100		49		49		X		144	
Total n (%)																
EMD	22,345 (100%)										11,319 (50.7%)		5904 (26.4%)		5122 (22.9%)	
EMD + RN	1158 (100%)										481 (41.5%)		306 (26.4%)		371 (32.0%)	

The proportion of calls dispatched with a priority in concordance with the ambulance personnel's assessment was calculated based on the defined corresponding priorities in Swedish Index and RETTS: Priority 1 corresponding to RETTS Red/Orange, Priority 2 corresponding to RETTS Yellow, Priority 3 corresponding to RETTS Green. Over- and undertriage were defined as the dispatched priority being higher or lower respectively than the priority as assessed by the ambulance personnel

Swedish Index, The Swedish Index to Emergency Medical Assistance; RETTS, The Rapid Emergency Triage and Treatment System; EMD, Emergency medical dispatcher; RN, Registered nurse

*(%) refers to the proportion of all calls dispatched by EMD (n = 22,345) and EMD + RN (n = 1158) respectively

EMD alone dispatched 11,319 (50.7%) of their calls with a priority in concordance with the ambulance personnel’s assessment, as compared to 481 (41.5%) for EMD + RN (*p* < 0.01). Overtriage was made in 26.4% of the calls, equal for both groups (EMD alone, n = 5904; EMD + RN, n = 306; *p* = 0.25) (Table 4). Of the overtriaged calls, the most adverse overtriage was made by EMD in 1,029 (17%) calls and by EMD + RN in 31 (10%) calls.

EMD undertriaged 5,122 (22.9%) of the calls as compared to 371 (32.0%) for EMD + RN (*p* < 0.01) (Table 4). Of the under-triaged calls, the most adverse undertriage was made in 305 (6%) and 44 (12%) of the calls by EMD alone and EMDs + RN, respectively.

### Sensitivity, specificity and predictive values

Sensitivity for the most urgent priority (Red/Orange) was 54.6% (95% CI: 53.6–55.6) for calls assessed by EMD as compared to 29.6% (25.1–34.5) for calls assessed by EMD + RN (*p* < 0.01), and specificity was 67.3% (95% CI: 66.5–68.1) and 84.8% (95% CI: 82.1–87.3) (*p* < 0.01) respectively (Table 5). Predictive values for the most urgent priority for calls assessed by EMD alone as compared to EMD + RN (respectively) were: PPV, 57.5% (95% CI: 56.5–58.5) and 49.4% (95% CI: 42.8–56.0) (*p* = 0.02); NPV, 64.6% (95% CI: 63.8–65.4) and 70.8% (95% CI: 67.7–73.7) (*p* < 0.01) (Table 5).

**Table 5:** Sensitivity, specificity, and predictive values for ambulance priorities

	EMD		EMD + RN		*p*-value
	Numbers	Probability % (95% CI)	Numbers	Probability% (95% CI)	
***Red/Orange***					
Sensitivity	5468/(5468 + 4544)	54.6% (53.6–55.6)	114/(114 + 271)	29.6% (25.1–34.5)	< 0.01
Specificity	8299/(4034 + 8299)	67.3% (66.5–68.1)	656/(117 + 656)	84.8% (82.1–87.3)	< 0.01
PPV	5468/(5468 + 4034)	57.5% (56.5–58.5)	114/(114 + 117)	49.4% (42.8–56.0)	0.016
NPV	8299/(4544 + 8299)	64.6% (63.8–65.4)	656/(271 + 656)	70.8% (67.7–73.7)	< 0.01
***Yellow***					
Sensitivity	5531/(5531 + 3583)	60.7% (59.7–61.7)	318/(318 + 186)	63.1% (58.7–67.3)	0.30
Specificity	7122/(6109 + 7122)	53.8% (53.0–54.7)	238/(416 + 238)	36.4% (32.7–40.2)	< 0.01
PPV	5531/(5531 + 6109)	47.5% (46.6–48.4)	318/(318 + 416)	43.3% (39.7–47.0)	0.03
NPV	7122/(3583 + 7122)	66.5% (65.6–67.4)	416/(318 + 416)	56.1% (51.3–60.9)	< 0.01
***Green***					
Sensitivity	320/(320 + 2899)	9.9% (8.9–11.0)	49/(49 + 220)	18.2% (13.9–23.5)	< 0.01
Specificity	18,243/(883 + 18,243)	95.4% (95.1–95.7)	745/(144 + 745)	83.8% (81.2–86.1)	< 0.01
PPV	320/(320 + 883)	26.6% (24.1–29.2)	49/(49 + 144)	25.4% (19.5–32.2)	0.80
NPV	18,243/(2899 + 18,243)	86.3% (85.8–86.7)	745/(220 + 745)	77.2% (74.4–79.8)	< 0.01

EMD, Emergency medical dispatcher; RN, Registered nurse; PPV, Positive predictive value; NPV, Negative predictive value; CI, Confidence interval

### Proportion of calls dispatched with a medical condition in concordance with the ambulance personnel’s assessment

Overall, the five most common dispatched medical conditions were “chest pain/cardiac disease” (n = 4185, 19.1%) “breathing difficulties” (n = 2912, 13.3%), “disease/injury to limbs—minor injuries” (n = 2708, 12.4%), “abdomen/urinary tract symptoms” (n = 2382, 10.9%) and “unspecific symptoms/acute severe disorders” (n = 2071, 9.5%) (Additional File 2).

EMD alone dispatched a medical condition in concordance with the ambulance personnel’s assessment in 13,785 (66.4%) of their calls as compared to 697 (62.2%) for EMD + RN (*p* = 0.01) (Table 6). Stratified by the five most commonly dispatched medical conditions, the proportion of calls dispatched with a medical condition in concordance with the ambulance personnel’s assessment ranged from 36.3 to 84.4% for EMD alone and 34.9 to 89.3% for EMD + RN. The proportions were higher for EMD alone compared to EMD + RN for each of these condition, with the exception of “abdomen/urinary tract symptoms” (Table 6). Irrespective of whether the assessment was made by EMD or EMD + RN, the highest concordance was demonstrated for calls dispatched as “abdomen/urinary tract symptoms” and the lowest concordance was demonstrated for “unspecific symptoms/severe disorders”.

**Table 6:** Dispatched medical condition in concordance with ambulance personnel's assessment; overall and stratified by most common conditions

	EMD	EMD + RN	*p*-value
	n = 20,761	n = 1120	
	n (%)	n (%)	
Over all	13,785 (66.4)	697 (62.2)	0.01

Stratified by the most common dispatched medical conditions	n (%)*	n (%)*	*p*-value
Chest pain/Cardiac disease	2993 (74.7)	116 (64.4)	0.01
Breathing difficulties	1836 (65.5)	47 (42.3)	< 0.01
Disease/injury to limbs/minor injuries	1677 (63.9)	46 (54.8)	0.09
Abdomen/Urinary tract symptoms	1838 (84.4)	183 (89.3)	0.07
Unspecific symptoms/severe acute disorders	706 (36.3)	44 (34.9)	0.76

EMD, Emergency medical dispatcher; RN, Registered nurse

*Refers to the proportion of all calls dispatched by each group respectively with the specified medical condition

## Discussion

Our results demonstrate that more precise emergency medical dispatching, measured as dispatch priority, accuracy, and medical condition, was not obtained when an EMD had the support of an RN. EMDs working without an RN dispatched most of the calls with the two highest priorities, while when supported by an RN, the proportion of Priority 1 calls was substantially lower. Concordance in the assessment of priority was higher between the EMD and ambulance when the calls were assessed by the EMD alone. Undertriage was observed more often in calls assessed by an EMD with support from an RN. Neither an EMD alone nor EMD supported by an RN performed the dispatch prioritization for calls that were assessed by the ambulance personnel to be of most urgent priority (Red/Orange) with a high sensitivity, that is, some of the most urgent cases were not identified as such. However, EMDs alone were more likely to do so when not supported by an RN. Conversely, the specificity was higher when the EMD was supported by an RN. Both groups had both similar and the highest sensitivity for patients that were assessed as Yellow by the ambulance personnel. Both groups had a low sensitivity with respect to the least urgent priority (Green); the EMD alone with half the sensitivity of that when supported by an RN.

### Proportion of calls dispatched with a priority in concordance with the ambulance assessment and levels of over/undertriage

At all priority levels, the dispatched priority was concordant with the ambulance assessment of priority more often when the call was handled by an EMD alone than when an EMD was supported by an RN. However, for both groups, the proportions reported were fairly low. In other Swedish settings, the overall concordance has been reported as 60% [29] and with a range of concordances when stratified per priority (Priority 1, 27%; Priority 2, 53%; Priority 3, 82%) [23].

Without regard to the concordance with ambulance personnel, an EMD alone dispatched at higher priority levels than when supported by an RN (in fact, more than 90% of a lone EMD’s calls were dispatched with the two highest priority levels). Specifically, when supported by an RN, an EMD’s calls were less frequently dispatched as Priority 1 and more frequently dispatched as Priority 3. These findings agree with those of Rawshani et al. who concluded that EMDs without a professional medical background had a higher consumption of pre-hospital resources [12]. The reported distribution of dispatched priority varied in previous Swedish studies [23, 29, 30], and the current study shows the same pattern for EMD as reported recently [26].

Precision in priority level is a challenge in emergency medical dispatch which is reflected in the amount of over- and undertriage found in the current study. Overtriage can be thought of as patient-safe, but also a non-optimal use of ambulance resources. In our study, both an EMD alone and an EMD with RN support had similar proportions of overtriage. The most adverse overtriage might be considered a more severe issue in terms of resource usage, which was observed more frequently with an EMD alone. Undertriage results in a delay in care, and an increased risk for patient deterioration while waiting for the ambulance [31]. The proportion of undertriage was higher when the EMD was supported by an RN than when not supported by an RN. Furthermore, EMDs supported by RN tended to perform the most adverse undertriage more frequently than EMDs alone.

At present, there are no guidelines about what levels of over- and undertriage are acceptable or desirable in emergency medical dispatch. The best comparison that can be made is with the international guidelines for trauma triage, which stipulate an acceptable range of overtriage between 25 and 35% and undertriage between 1 and 5% [32]. The proportion of overtriage calls seen in this study might thus be acceptable, while the proportion of undertriage seen in both groups was substantially higher than these guidelines recommend.

### Sensitivity, specificity and predictive values

Overall, the magnitude of sensitivity reported in this study for both groups and all priorities can be considered low. EMD alone had a higher sensitivity and PPV for the most urgent priority (Red/Orange), while EMD supported by RN had a higher sensitivity for the least urgent priority (Green). This result corroborates previous Swedish findings that among patients presenting with chest pain, EMDs with a medical professional background were at greater risk of not identifying life-threatening cases [12]. In a study restricted to helicopter assisted emergencies in UK, the introduction of EMDs without a medical professional background, assisted by an additional triage algorithm, was associated with a more accurate dispatch [13].

Comparing the rates of predictive values between and within dispatch systems requires knowledge about the prevalence of severity levels found in the field (i.e., detected by the reference standard) [33]. Therefore, it is important to note that the prevalence of the most urgent priorities (patients assessed as RETTS Red/Orange by the ambulance) was not entirely similar among the calls assessed by each group (45% for EMD and 33% for EMD supported by RN).

In a previous study on the Swedish Index, the sensitivity for the most urgent priority was 94.5% [30]. However, that test applied a different positivity cut-off than this study (Swedish Index Priorities 1 and 2 corresponded to RETTS Red/Orange/Yellow). Red and Orange both indicate urgent conditions, with the difference being “immediate” or “potentially” life threatening. On the other hand, Yellow is clearly defined as a “non-life-threatening” condition [17]. Red and Orange share more common features in relation to urgency and need for prompt and urgent medical assistance than the others, which was the argument for the current definition on corresponding priorities.

International data on sensitivity for time-critical and severe calls has been reported to be as high as 93.3% and 86–87.5% for Australia and Switzerland respectively [33–35]. The sensitivity for cardiac arrest has been studied for different settings and reported to range from 65.9 to 86% in various countries [36–39]. As for levels of over/undertriage, there is no consensus on desirable levels of sensitivity, specificity, and predictive values [40, 41]. Nevertheless, given the magnitudes of these values in the current study, it seems reasonable to conclude that the performances of both EMDs alone and EMDs supported by RNs leave room for improvement.

### Proportion of calls dispatched with a medical condition in concordance with ambulance assessment

The dispatch of a medical condition in concordance with the ambulance assessment was seen more often in calls assessed by an EMD alone. The difference, though statistically significant, might not be clinically relevant (66.4% *vs* 62.2%). Even though precision for priority can be considered as the overriding factor in emergency medical dispatch, an assessment of the medical condition of the patient is valuable for several reasons. Based on the medical condition described, the EMDs and RNs give instructions for callers to follow until an ambulance arrives [42, 43], and meanwhile the ambulance crew prepare itself based on the dispatch information [44]. Ambulances staffed with highly specialized crews and equipment are dispatched for a subset of calls that are medical, surgical, and traumatic [45–47], which require an understanding of the medical condition. One could argue that when an RN supports the EMD, a higher precision for the medical condition should be expected. This study found no such a difference, except for the tendency seen in calls dispatched as “abdomen/urinary tract symptoms”. The dataset does not allow us to know the reported symptoms for each call (although the five most frequently dispatched medical conditions were similar for both groups). Previously, EMDs have described “the complicated, somewhat diffuse, calls” to be the most difficult to assess [48]. At the time of this study, there were no internal guidelines about when the EMD should request RN support. Hence, factors other than the reported symptoms could also have affected the EMDs decision to request RN support. Such factors could include, e.g., call load (i.e., the number of simultaneously incoming 112 calls), the access to an available RN at the time of the call, and individual characteristics of the EMD.

### The complexity of the emergency medical dispatch decision-making process

The required level of education of EMDs, including their professional background, has been identified as an area in need for further research [8]. In general, medical training is not required of an EMD in Sweden. However, some regions do operate their own dispatch centers, manned only by RNs [7]. The complex task of emergency medical dispatching requires expertise in time-efficient communication and the ability to identify the urgency of the call, and to be able to determine the appropriate resources required at the right time, as well as creating an alliance with the caller and providing adequate pre-arrival instructions [49]. Incorrect dispatch decisions have been associated with second-hand information provided by people other than the affected person, callers describing the problem insufficiently, and insufficient interrogation of the caller [50–52]. The current findings of differences between the two groups in precision of dispatch decision could potentially be explained by such factors. Moreover, the results could also be recalled by variations in adherence to the dispatch protocol. Variation in adherence to dispatch protocol has been reported among RNs at Norwegian EMCCs [53, 54]. There is no similar data on level of protocol adherence regarding random calls from a Swedish setting. In a qualitative study, adherence to the protocol was acknowledged by RNs at the EMCC as important, but in situations of difficult decisions, other criteria, particularly intuition, was emphasized [55]. How EMDs and RNs deal with the decision-making process, including the level of protocol adherence, is of great interest, however, this topic is beyond the scope of the current study.

### Challenges when reporting on and evaluating the outcome of emergency medical dispatch

Comparisons among dispatch systems, and conclusions about what systems might be superior, are impeded by discrepancies in dispatch protocols and different reference standards used in the literature [40, 41]. In the Swedish setting, the current data is unique, in that linkage of data between the medical dispatching and the ambulance service is not routinely performed outside of the described project. However, it is important to understand that comparing dispatch assessment with ambulance assessment (as done in the current study) is not straightforward. Unexpressed information about the situation during the call might result in a significant difference between the two assessments. Moreover, additional events may occur after the call but before the ambulance arrives at the scene. Hence, calling a dispatch decision “correct” or not is not entirely appropriate, because the true condition of the patient is not known at the time of the call. Furthermore, the performance of the reference standard, RETTS, in predicting high acuity patients as Red or Orange in the pre-hospital setting has been found as being over 80%, with a lower sensitivity in elderly, and a corresponding 64% specificity [26]. Although, as the same reference was used for both groups, it will not impact on the results. Taken together, a consensus of the main objective for emergency medical dispatching, and how this should be evaluated is needed. A starting point could be the linking of dispatching, prehospital and hospital data sets, which would allow for an analysis of patient-related outcomes. Moreover, the lack of common standards in reporting on precision and accuracy in emergency medical dispatching has been highlighted [40]. The magnitude and clinical implication of the current results aside, it is a strength to report on several outcomes. To enable comparison, the reporting in the current study has been made in accordance with the standards for the reporting of diagnostic accuracy studies when possible [56].

## Limitations

The current study has some limitations which need to be acknowledged. The length of work experience and additional background characteristics (such as age and gender) of the EMDs, RNs, and callers was unknown. The time of day for the calls was not accounted for in the analysis, a factor that has previously been shown to be associated with differences in the odds of identifying fatal and life-threatening conditions [12]. Another limitation is that the two groups compared (calls with an EMD alone and calls with EMD supported by an RN) were of quite unequal sizes, which can be partially explained by the fact that, in 2015, SOS Alarm employed 463 EMDs and 37 RNs. Several calls had to be excluded because of missing data and inconclusive ambulance assessments. To err on the side of caution, ambulances reporting their assessment as being “same assessment as the dispatcher” were excluded, because it was unclear whether that conclusion was based on the dispatched priority level or the medical condition. There is no established practice, nor were there instructions in the quality improvement project, regarding whether priority levels should correspond between the Swedish Index and RETTS. As an aside, a strength of this study is that the final cohort included a large diversity of unselected calls, rather than being restricted to calls about a specific medical condition; such “random” calls have been used as the primary data to a little extent previously in the Swedish context; [23, 29, 30].

Finally, the choice of reference standard in this study may restrict the generalizability of the results. Naturally, the ambulance personnel were informed of the dispatched priority level and medical condition of each patient, which might have affected their assessments and consequently the outcome measures in the study. Using the ambulance personnel’s immediate assessments of the patients was considered to best reflect the urgency and nature of medical condition at the time when the emergency medical call was made. The assessment was made before further care was given to the patient, to prevent the influence of medical interventions. There is no data available on whether ambulance personnel deviated from adherence to RETTS in their assessments. Such deviation could affect the results. However, the use of RETTS to assess patients is part of the ambulance personnel’s’ routine work and is included in their education and training.

## Conclusion

Concordance with the ambulance personnel’s assessment, both with respect to dispatch priority and medical condition, were higher in calls assessed by an EMD alone. Furthermore, calls assessed by an EMD alone were less likely to be undertriaged and were also more likely to identify the most urgent cases. However, when supported by an RN, the specificity for the most urgent cases was superior. Overall, higher precision of emergency medical dispatching was not observed when the EMD was supported by an RN. Symptoms reported by the caller were unknown and could potentially recall for the difference between the groups. Nevertheless, equality in nature of callers’ conditions is indicated by the result that the five most frequently dispatched medical conditions were similar for both groups. How patient safety is affected by the observed divergence in dispatched priorities is an area for future research. Moreover, a consensus of the main objective for emergency medical dispatching, and how this should be evaluated is needed. A starting point could be the linking of dispatching, prehospital and hospital data sets, which would allow for an analysis of patient outcomes.

## Supplementary Information

**Additional file 1.** Definition of corresponding medical conditions in Swedish Index and RETTS.

**Additional file 2.** Distribution of dispatched medical condition, over all and stratified by calls assessed by emergency medical dispatcher (EMD) and EMD supported by registered nurse (EMD + RN).

## Data Availability

The datasets used and analyzed during the current study are available from the corresponding author upon reasonable request.

## Abbreviations

EMD: Emergency Medical Dispatcher
RN: Registered nurse
EMCC: Emergency Medical Communication Center
Swedish Index: The Swedish Index to Emergency Medical Assistance
RETTS: The Rapid Emergency Triage and Treatment System
ESS: Emergency Signs and Symptoms
