# Supplementary material for: A registry-based observational study comparing emergency calls assessed by emergency medical dispatchers with and without support by registered nurses
Source: Scand J Trauma Resusc Emerg Med. 2022 Jan 10;30:1. doi: 10.1186/s13049-021-00987-y (PMC8744325; doi:10.1186/s13049-021-00987-y)
Supplement: Supplementary file 1 — Additional file 1. Definition of corresponding medical conditions in Swedish Index and RETTS. [file 13049_2021_987_MOESM1_ESM.docx]

| **Additional file 1.** Definition of corresponding medical conditions in Swedish Index and RETTS. | |
| --- | --- |
| Swedish Index | RETTS, ESS |
| Allergy | Allergy/Drugs |
| Breathing difficulties | Dyspnea/Hyperventilation |
| Bleeding, non traumatic | Gastrointestinal bleeding or Bleeding ear/nose/throat |
| Scorch/electrical induced disorders or Chemical/gas induced disorders | Scorch/chemical/electrical induced disorders |
| Chest pain/Cardiac disease | Chest pain or Arythmia/Atrial fibrilation or High/low blood pressure |
| Abdomen/Urinary tract symptoms | Abdominal pain/nausea/diarrhea or Genital disorders, Men or Urinary tract disorders or Genital, women/pregnancy |
| Diabetes | Diabetes/hyperglaucemia or Hypoglucemia |
| Extremity/wound and injuries/minor trauma | Extremity disorders or Hand/arm/shoulder or Hip/knee/foot |
| Fever | Infection/fever |
| Intoxication, overdose OR Child- intoxication | Intoxication |
| Gynecology-pregnancy (before w.20) or Pregnancy/delivery (from w.20) | Genital, women/pregnancy |
| Headache, vertigo | Headache or Vertigo/diziness |
| Seizure | Seizure/epilepsy/febrile seizure |
| Accident (Trauma) | Head/cervical/neck or Thorax/Back/Abdomen |
| Unspecific symptoms/severe acute disorders | Unspecific symptoms or Syncope or Vertigo/diziness |
| Back disorders | Backpain |
| Stroke - paralysis | Stroke/Transient Ischemic Attack |
| Suspected suicide - psychiatri | Psychiatric disorders |
| Violence/abuse | Abuse |
| Eyes-ear-nose-throat | Bleeding ear/nose/throat or Foreign body/nose/respiratory tract or Faryngotonsillitis or Injury/infection, eye or Ear disorders |
| The ambulance personnel’s assessment of the medical condition, according to RETTS ESS, was used as the reference standard in the analyses on proportion of calls dispatched with a medical condition in concordance with the ambulance personnel’s assessment. The corresponding medical conditions in the Swedish Index and RETTS were defined prior to analysis as shown above. Non-corresponding dispatched medical conditions (dispatched medical conditions according to Swedish Index for which no appropriate RETTS ESS were deemed to correspond): Child-disease, Animal/Insect-bite, Hypo/Hyperthermia, Danger of life, Child-unconscious, Adult unconscious, Suspected health service demand. | |
| Abbreviations: "Swedish Index to Emergency Medical Assistance" (Swedish Index), "The Rapid Emergency Triage and Treatment System" (RETTS), "Emergency Signs and Symptoms" (ESS). | |
