# Supplementary material for: A registry-based observational study comparing emergency calls assessed by emergency medical dispatchers with and without support by registered nurses
Source: Scand J Trauma Resusc Emerg Med. 2022 Jan 10;30:1. doi: 10.1186/s13049-021-00987-y (PMC8744325; doi:10.1186/s13049-021-00987-y)
Supplement: Supplementary file 2 — Additional file 2. Distribution of dispatched medical condition, over all and stratified by calls assessed by emergency medical dispatcher (EMD) and EMD supported by registered nurse (EMD + RN). [file 13049_2021_987_MOESM2_ESM.docx]

| **Additional file 2. Distribution of dispatched medical condition, over all and stratified by calls assessed by emergency medical dispatcher (EMD) and EMD supported by registered nurse (EMD + RN)** | | | |  |
| --- | --- | --- | --- | --- |
| **Dispatched medical condition, Swedish Index** | **Over all n= 21 881 n (%)** | **EMD n= 20 761 n (%)** | **EMD + RN n= 1 120 n (%)** |  |
| Chest pain/Cardiac disease | 4185 (19,1) | 4005 (19.3)¹ | 180 (16.1)² |  |
| Breathing difficulties | 2912 (13,3) | 2801 (13.5)² | 111 (9.9)⁴ |  |
| Disease/injury to limbs-minor injuries | 2708 (12,4) | 2624 (12.6)³ | 84 (7.5) |  |
| Abdomen/Urinary tract symptoms | 2382 (10,9) | 2177 (10.5)⁴ | 205 (18.3)¹ |  |
| Unspecific symptoms/severe acute disorders | 2071 (9,5) | 1945 (9.4)⁵ | 126 (11.3)³ |  |
| Stroke - paralysis | 1691 (7,7) | 1629 (7.8) | 62 (5.5) |  |
| Headache, vertigo | 1284 (5,9) | 1190 (5.7) | 94 (8.4)⁵ |  |
| Accidents (Trauma) | 795 (3,6) | 784 (3.8) | 11 (0.1) |  |
| Fever | 657 (3) | 603 (2.9) | 54 (4.8) |  |
| Back disorders | 578 (2,6) | 548 (2.6) | 30 (2.7) |  |
| Seizure | 491 (2,2) | 481 (2.3) | 10 (0.9) |  |
| Diabetes | 471 (2,2) | 443 (2.1) | 28 (2.5) |  |
| Bleeding, non trauma | 451 (2,1) | 430 (2.1) | 21 (1.9) |  |
| Intoxication, overdose | 409 (1,9) | 378 (1.8) | 31 (2.8) |  |
| Suspected suicide - psychiatri | 262 (1,2) | 241 (1.2) | 21 (1.9) |  |
| Allergy | 169 (0,8) | 155 (0.7) | 14 (1.3) |  |
| Gynecology-pregnancy (before w.20) | 116 (0,5) | 94 (0.5) | 22 (2.0) |  |
| Violence/abuse | 103 (0,5) | 101 (0.5) | 2 (0.2) |  |
| Pregnancy/delivery (from w.20) | 76 (0,3 ) | 65 (0.3) | 11 (1.0) |  |
| Eyes-ear-nose-throat | 44 (0,2) | 43 (0.2) | 1 (0.1) |  |
| Scorch/electrical induced disorders | 19 (0,1) | 17 (0.1) | 2 (0.2) |  |
| Child- intoxication | 5 (<0.01) | 5 (<0.01) | 0 |  |
| Chemical/gas induced disorders | 2 (<0.01) | 2 (<0.01) | 0 |  |
| Abbreviations: Emergency medical dispatcher (EMD), Registered nurse (RN), "The Swedish Index to Emergency Medical Assistance" (Swedish Index). ¹²³⁴⁵ The superscripted numbering indicates the five most frequently dispatched medical conditions, for EMD and EMD + RN respectively. | | | |  |
|  |  |  |  |  |
